# High-frame rate four dimensional optoacoustic tomography enables visualization of cardiovascular dynamics and mouse heart perfusion

**DOI:** 10.1038/srep10133

**Published:** 2015-07-01

**Authors:** Xosé Luís Deán-Ben, Steven James Ford, Daniel Razansky

**Affiliations:** 1 Institute for Biological and Medical Imaging (IBMI), Helmholtz Center Munich, Ingolstädter Landstraβe 1, 85764 Neuherberg, Germany; 2 Faculty of Medicine, Technical University of Munich, Ismaninger Straβe 22, 81675 Munich, Germany.

## Abstract

Functional imaging of mouse models of cardiac health and disease provides a major contribution to our fundamental understanding of the mammalian heart. However, imaging murine hearts presents significant challenges due to their small size and rapid heart rate. Here we demonstrate the feasibility of high-frame-rate, noninvasive optoacoustic imaging of the murine heart. The temporal resolution of 50 three-dimensional frames per second provides functional information at important phases of the cardiac cycle without the use of gating or other motion-reduction methods. Differentiation of the blood oxygenation state in the heart chambers was enabled by exploiting the wavelength dependence of optoacoustic signals. Real-time volumetric tracking of blood perfusion in the cardiac chambers was also evaluated using indocyanine green. Taken together, the newly-discovered capacities offer a unique tool set for *in-vivo* structural and functional imaging of the whole heart with high spatio-temporal resolution in all three dimensions.

Human cardiac diseases are linked to many inheritable and environmental factors, but the link between molecular- and organ-level dysfunction remains unclear. As more primary causes of cardiovascular disease are identified, the need to characterize their functional effects and therapeutic responses is growing. Many transgenic and surgical mouse models have been developed to better understand a wide variety of cardiac diseases identified in humans, such as models of inheritable cardiomyopathies, diseases related to chronic ventricular afterload, myocardial infarction^1^ and diabetes^2^. As a result, much of our understanding of the mechanisms underlying human cardiac health and disease come from studies of the murine heart. However, delineating correlations and causations of cardiac disease requires investigating the link between the molecular cascade of excitation-contraction and organ-level function, preferably in studies of the same heart.

Charaterization of cardiac function in such models is often performed *ex vivo*, utilizing the Langendorf perfusion method^3^ among many other *in vitro* and *in situ* methods. Relatively recent developments in biomedical imaging have allowed for *in-vivo* characterization of organ-level function by cardiac ultrasound^4^,^5^,^6^,^7^ and cardiac magnetic resonance imaging (MRI)^8^,^9^,^10^. Such methods have been readily applied in humans to characterize cardiac functional parameters, e.g. cardiac volume and ejection fraction. However, cardiac imaging in small animals using conventional clinical imaging modalities remains challenging due to the small size and rapid heart rates reaching 400–600 beats per minute (bpm) in mice. Ultrasound (US) imaging, although prone to intraobserver variability, is arguably the most advanced approach for fast cardiac imaging in small animals. Indeed, internal structures of the heart can be identified in US images, whereas other important physiological parameters such as blood flow, myocardial fiber orientation and elasticity or electrically-induced deformations can also be coregistered with structural images 11,12,13. More importantly, ultrafast US imaging was made feasible by advancements in processing methods, recently demonstrating utility for pulse-echo, Doppler and shear-wave imaging of the heart in three-dimensions^14^. On the other hand, cardiac MRI is still limited by significantly lower temporal resolution and volumetric scans require image acquisition over multiple cardiac cycles. As a result, MRI systems cannot capture high spatio-temporal resolution information at all phases of the cardiac cycle even in humans where heart rate is much slower than in mice. In addition, gating techniques and post processing must be further used to artificially enhance the temporal resolution of the images and reduce motion-related artifacts.

The small size of the mouse heart additionally requires a high-resolution imaging platform to provide robust volumetric analyses. *In-vivo* imaging at high spatial resolution can be achieved by magnetic resonance microscopy (MRM), which can be used to visualize even small embryonic mouse hearts^15^,^16^,^17^. However, MRM is limited by slow acquisition times, and does not have the ability to resolve probes with contrast comparable to optical techniques. Confocal/nonlinear microscopy techniques can be used instead to resolve molecular probes with excellent spatial resolution, contrast and specificity. Yet, due to intense light scattering and lack of imaging depth, microscopy techniques require invasive imaging strategies^18^,^19^,^20^, or *ex-vivo* (e.g. Langendorf) methods to reach the heart. Lastly, optical coherence tomography methods can also provide high spatio-temporal images in 4D, but are similarly limited to surface-level investigations^21^ or otherwise studies on small-sized embryonic hearts^22^,^23^,^24^,^25^.

Optoacoustic imaging holds multiple prospective advantages for cardiac imaging as it: (1) provides high spatial and temporal resolution; (2) possesses optical contrast, and hence can be used in combination with a large selection of optical contrast agents to target molecular level dysfunction, and; (3) has the advantage of deeper penetration, allowing for imaging of cardiac function truly *in*-*vivo* and in an intact heart. By relying on detection of light-induced pressure variations, optoacoustics benefits from both high spatio-temporal resolution of ultrasound and contrast-related advantages of light illumination^26^. 2D optoacoustic imaging of murine cardiovascular dynamics at a frame rate of 10 Hz was previously reported by using curved arrays of ultrasonic detectors^27^. By means of multispectral optoacoustic tomography (MSOT) it was further shown possible to target non-invasively myocardial infarction (MI) using specific spectral absorption signature of a dendritic polyglycerol sulfate-based (dPGS) near-infrared imaging agent targeted to P- and L-selectins^28^. Cross-sectional (2D) imaging approaches result in strongly anisotropic and non-uniform spatial resolution manifested in the so-called “out-of-plane artifacts”, image blurring and overall deterioration of image quality and contrast. Furthermore, volumetric (3D) rendering might only be achieved in this case via translation and stacking of multiple cross-sectional images, which cannot be done in real time. This inevitably leads to further loss of image quality and difficulties in quantification due to motion artifacts, which are exacerbated by the fast dynamic motion of the heart.

Here we demonstrate, for the first time to our knowledge, optoacoustic visualization of fast beating mouse heart in three dimensions rendered at a high frame rate of 50 volumetric images per second. Furthermore, by sequential acquisition of data at several excitation wavelengths, we were able to differentiate blood oxygenation levels in the heart chambers based on spectral dependence of the optoacoustic data. Finally, high-temporal-resolution tracking of blood flow in the heart has been made possible by visualizing the distribution of intravenously-administered indocyanine green optical contrast agent.

## Results

### Three-dimensional imaging of the murine cardiac cycle

**Figure 1:**
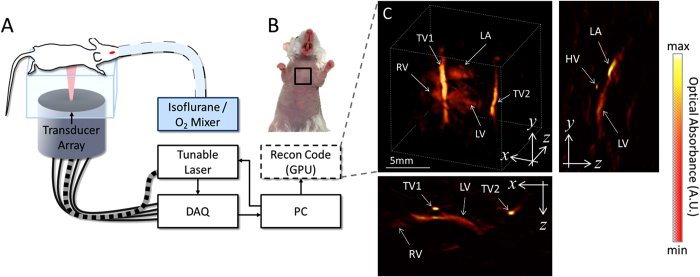
Experimental layout. The experimental setup of the 4D optoacoustic tomography system is shown in panel **A**. The mouse was oriented in a prone position over the active surface of the ultrasound detector array. The heart of the mouse was illumated by shining laser light upon the chest via a fiber bundle connecting the tunable laser output to the center of the detector array. The imaged region corresponding to the live mouse heart is illustrated by the black square in panel **B**. A volumetric reconstruction of the heart and surrounding vessels is showcased in panel **C**. The cross-sections corresponding to the central position of the represented three-dimensional volume are also displayed in panel C. Important features of the heart, including the right ventricle (RV), left ventricle (LV), and left atrium (LA) are prominently visualized using the optoacoustic imaging approach. Additionally, two thoracic vessels (TV1 and TV2) can be clearly seen in the vicinity of the heart as well as a heart vessel (HV) indicated in a cross-section. The mouse and setup illustrations in A were developed by the authors.

For *in vivo* imaging, the mouse was placed in a prone position over the active surface of the ultrasound detector array, as indicated in Fig. 1a. Further details on the mouse handling and experimental setup are provided in the online methods section. A representative volumetric (three-dimensional) optoacoustic image of the heart region of the chest (marked in Fig. 1b) is shown in Fig. 1c along with the sagittal and transverse cross-sections made approximately through the center of the heart. Important physiological structures, including the right ventricle (RV), left ventricle (LV), and left atrium (LA), as well as two thoracic vessels (TV1 and TV2), are prominently visualized in Fig. 1c. Clearly, the image quality is affected by several factors. First, relatively large absorbing structures, such as the heart, would emit predominantly low frequency optoacoustic responses that cannot be efficiently detected using limited-bandwidth transducers. In this work, detectors with approximately 100% bandwidth around a central frequency of 4MHz were used, which would mainly recover optoacoustic signals originating from submillimeter structures. Second, the so-called limited-view effects caused by the incomplete angular coverage (90^o^ solid angular coverage of the spherical array probe in our case) also affect the visibility of the lateral sides of the heart. Finally and importantly, the highly absorbing blood in the heart chambers causes significant light attenuation, leading to strong bias toward structures at the myocardium surface. Each of these factors contributed to the limited volume of the heart which can be efficiently visualized with our optoacoustic system.

**Figure 2:**
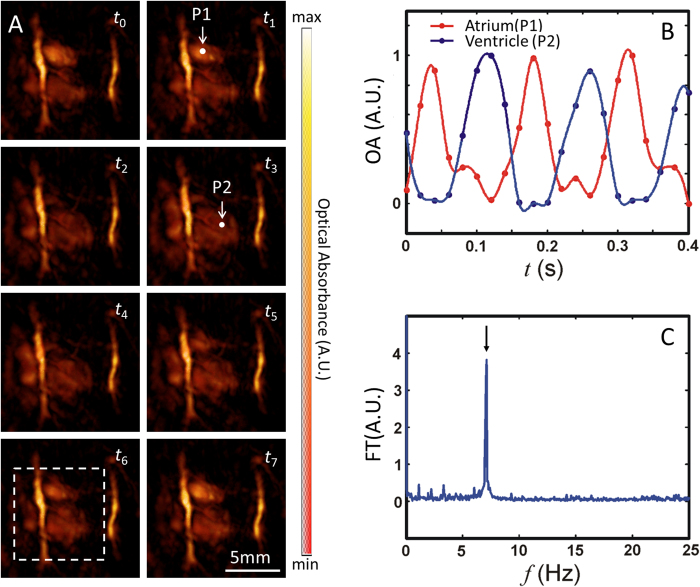
>Cardiac dynamics visualized by high temporal resolution single-wavelength illumination. Images of the heart at single-wavelength illumination (800 nm) are shown for a series of eight consecutive frames (*t*_0_–*t*_7_, 20 ms time steps) in panel **A**. Points of interest are indicated on the left atrium and ventricle as P1 and P2, respectively. Because absorbance at 800 nm reflects the total blood volume, the change in signal at P1 and P2 reflects changes in the blood volume in the LV and LA, respectively. This time-dependent change in signal (normalized) is shown in panel **B** for P1 (Atrium, red) and P2 (Ventricle, blue), where points reflect data and traces indicate spline fits. The mean temporal Fourier Transform (FT) of a subvolume of data (indicated by the dashed white square in panel **A**) is shown in panel **C**. The peak in panel **C** indicated by an arrow correlates to the frequency of the signal induced by the heart beat.

The potential for visualizing fast cardiac cycle dynamics was demonstrated by acquisition of optoacoustic images at 50 Hz. Fig. 2a represents volumetric images of eight consecutive time points, acquired within one mouse cardiac cycle. The images are represented as maximum intensity projections (MIP) along the *z* direction for the three-dimensional data. In an attempt to quantify cardiac dynamics, the optoacoustic signal was traced over time at two locations, corresponding to the left atrium and left ventricle, indicated respectively by P1 and P2 in Fig. 2a. Fig. 2b showcases the time profiles of the optoacoustic signals at P1 and P2, as indicated by red and blue data traces, respectively. Cardiac dynamics can also be qualitatively assessed in the movies accompanying the manuscript. Movie 1 showcases a three-dimensional rotating view of a beating heart imaged at 800 nm. Note that the frame rate was slowed down to 25 Hz to optimize the visual perception of fast motion. In this movie, respiratory cycles can also be perceived along with the cardiac cycles. From this data, the heart rate can be estimated by approximating the Fourier Transform (FT) of the time profiles. Displayed in Fig. 2c is the mean FT of the time profiles for the image voxels within the dashed square indicated in Fig. 2a – calculated from the whole sequence of 1000 acquired frames at 800 nm. This curve shows a prominent peak at approximately 7.1 Hz, which corresponds to a heart rate of around 426 bpm. Spectral components at lower frequencies were not as prominently featured in the data, but could be possibly attributed to breathing-related motion. Overall, the heart rate measured in the FT domain represents the expected values in adult mice subjected to isoflurane anesthesia^29^.

### Blood oxygenation state of cardiac chambers

**Figure 3:**
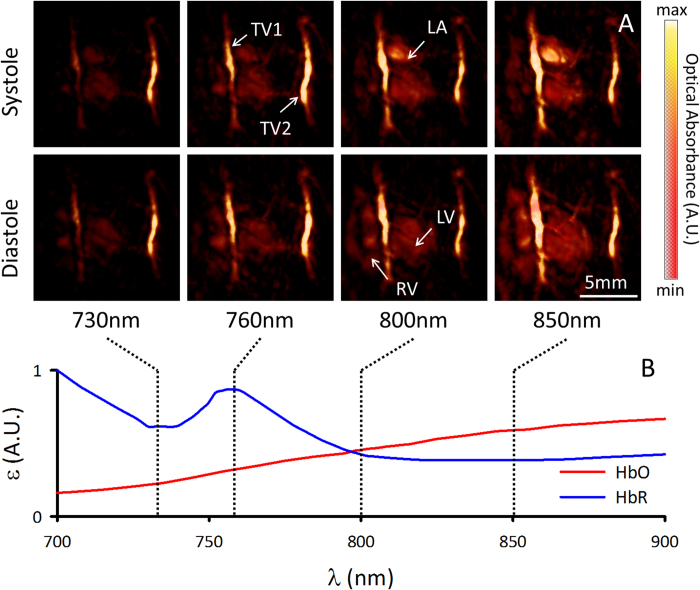
Functional information derived from multispectral volumetric cardiac images. Images of the heart at systole and diastole are shown in panel **A** for each of the 730, 760, 800, and 850 nm acquisition wavelengths. The images are represented as maximum intensity projections along the depth axis, and intensities were normalized to the energy of the laser for each wavelength. The difference in the absorption profile of oxygenated and deoxygenated blood are respectively indicated by red (HbO, oxidized hemoglobin) and blue (HbR, reduced hemoglobin) lines in panel **B**. The wavelengths selected for experimentation are indicated by vertical dashed lines, and allowed for the distinction of relative oxygenation levels in the heart. Prominent anatomical features seen in Fig. 1c (right ventricle (RV), left ventricle (LV), left atrium (LA), thoracic vessels TV1 and TV2) are also indicated in panel **A**.

Figure 3a shows optoacoustic images acquired at four different wavelengths, namely, 730, 760, 800, and 850 nm. Oxygenated and deoxygenated blood can be distinguished based on their different spectral behavior (Fig. 3b). As a rule of thumb, optoacoustic signal intensity increases with wavelength for cardiac chambers and other structures containing highly oxygenated blood (i.e., left atrium and left ventricle). For instance, the regions labeled LA and TV1 in Fig. 2a markedly increase their optical absorption with wavelength. Movie 2 shows the sequence of images at the four excitation wavelengths corresponding to several heart cycles in between the breathing events. Note that, due to the multi-wavelength illumination mode, Movie 2 is displayed at a reduced frame rate of 10Hz. The trend of increased optical absorbance at the higher wavelengths in the LA and TV1 regions is also evident in Movie 2, correlating well with the spectrum of oxygenated hemoglobin (Fig. 3b) and indicating that LA and TV1 contain highly-oxygenated blood. Thus, while anatomical and hemodynamic information is available through single-wavelength image acquisition, additional functional information can be retrieved when analyzing the multi-wavelength data.

### Real-time volumetric tracking of blood perfusion in the cardiac chambers

**Figure 4:**
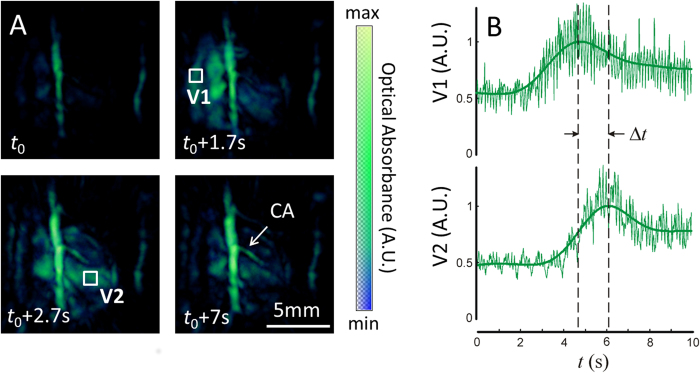
Tracking cardiac flow dynamics using blood-pool contrast agent, indocyanine green (ICG). Snapshots from the time series of images taken during the ICG injection experiment are shown as maximum intensity projections in panel **A**. Time points are chosen illustrating a baseline image at 800 nm (*t*_0_), the entry of the ICG bolus into the RV (*t*_0_ + 1.7s), later entry of ICG into the LV (*t*_0_ + 2.7s), and late phase, near homogenous distribution of ICG in the bloodstream (*t*_0_ + 7s). Volumes of interest are depicted on the maximum intensity projections corresponding to the right ventricle (V1) and left ventricle (V2), respectively. The intensities of the absorption signals at 800 nm for V1 and V2 are plotted in panel **B**. Peak values of the signal V1 and V2 represent the arrival times of ICG into the right and left ventricles, respectively. The difference in the time to peak values at V1 and V2 (Δ*t*) can therefore be used as a quantitiative measure of pulmonary transit time.

Volumetric tracking of blood perfusion was showcased with injection of indocyanine green (ICG) as described in the methods section. Four representative time instants corresponding approximately to the same phase of the cardiac cycle are shown in Fig. 4. The perfusion of the agent can be best perceived in a video file available in the on-line version of the journal (Movie 3), which is displayed at 25 Hz. In Fig. 4b the maximum absorption values for two volumes of interest (VOI) in the right ventricle (V1) and left ventricle (V2) are plotted as a function of time. The high-frequency fluctuations in the signal are mainly attributed to heart-beat and breathing-associated motion, which are larger than per-pulse variations of the laser energy, whereas the moving average readily reveals the slower high-magnitude signal variations in response to the changes in concentration of ICG. An increase in absorption is first perceived in the right side of the heart (left side of the image), whereas the maximum absorption in the left side of the heart (right side of the image) is delayed by several cardiac cycles. As shown in Fig. 4b, the difference in the time (Δ*t*) to peak values of ICG absorbance in the RV and the LV can be estimated. For this, 1000 different VOIs in the right and left ventricles were considered. An average value of Δ*t* = 1.24 seconds for all VOIs was subsequently obtained, where the uncertainty in Δ*t* amounted to a standard deviation of *s*(Δ*t*) = 0.35 seconds. Physiologically, this parameter describes the time it takes for blood to travel through the pulmonary circulation (from the RV to the LV) and is referred to as pulmonary transit time.

## Discussion and Conclusions

The results presented herein showcased the unique capability of four-dimensional optoacoustic tomography for studies of cardiac dynamics with high spatio-temporal resolution necessary for characterizing the fast beating mouse heart *in vivo*. Versatility of the system in resolving functional and, potentially, molecular contrast is enabled via fast acquisition of images at multiple excitation wavelengths. In this way, the basic cardiac functional analyses were derived here from the intrinsic spectral dependence of oxy- and deoxy-hemoglobin as well as extrinsically-administered ICG. The heart chambers were distinguished based on the basic analysis of spectral dependence of the optoacoustic signals in the respective regions of interest. Determination of additional intrinsic functional parameters, such as blood oxygen saturation levels, would necessitate development of more sophisticated processing methods to efficiently register images at the different wavelengths and accurately account for the wavelength-dependent light fluence distribution across the moving heart.

In this work, we additionally demonstrated high temporal-resolution tracking of blood flow in the heart by visualizing the distribution of ICG following intravenous injection. Due to the adequate penetration of optoacoustics, these measurements were possible non-invasively, without the need to open the chest of the animal. The pattern of blood flow determined in the ICG experiment was consistent with the expected blood flow within the heart, where blood first enters the right chambers and subsequently reenters (after circulating through the lungs) the left chambers of the heart. The dynamic imaging of blood circulating through the heart may allow for a better understanding and diagnosis of cardiovascular disease. One potential application would be diagnosis of ventricular dysfunction, which would be expected to affect the delivery of ICG signal from the right to the left ventricle. In the current study, we were able to resolve the pulmonary transit time, which may serve as a useful indicator of ventricular dysfunction, although further studies are required to determine whether the changes in this parameter in health and disease surmount the uncertainties in the measurements. Such an approach could also potentially characterize left ventricular dysfunction as a delay in the ICG signal within the LV, and accumulation of the ICG signal in the pulmonary veins. ICG signal within the lungs may further serve as an indicator of cardiogenic pulmonary edema, allowing for diagnosis and characterization of the disease progression. Another value may be found in the characterization of cardiac valve function, where, as an example, a slow but periodic increase in the ICG signal from the LV might indicate a leaky aortic valve. As a result, this system indicates the potential for use of optoacoustics as a hand-held clinical imaging system for the human cardiovascular system, though its applicability will mainly depend on the technical progress with achieving deeper optical penetration through highly absorbing blood. In this regard, improvements in the probe design and light delivery strategies may greatly benefit the clinical translation efforts of the cardiac optoacoustic imaging technology. Furthermore, other externally-administered contrast agents can be potentially targeted to molecular events to characterize underlying mechanisms of cardiac diseases. In the pre-clinical setting, such approaches would be useful in determining the efficacy of treatments of heart disease, e.g. myocardial-infarction-associated inflammation, cell death, or remodeling of the myocardium. Finally, optoacoustic imaging at high temporal resolution may have other physiological applications, such as the determination of ventricular function and hemodynamic analysis.

From the technical point of view, the presented 3D tomographic configuration provides nearly isotropic three-dimensional spatial resolution in the range of 200 μm^30^, though higher resolution can be rendered by capturing optoacoustically-induced responses at higher frequencies. Three-dimensional tomographic acquisition provides obvious advantages in terms of quantification performance and overall image quality over previously published results using cross-sectional (2D) optoacoustic imaging systems, which are generally prone to out-of-plane and motion-related artifacts when applied for volumetric imaging^31^,^32^. Yet, the lungs, located around the imaged heart region, require closer attention as air-filled cavities may generally produce strong acoustic attenuation and scattering, further leading to significant distortion in the reconstructed images^33^,^34^. In this regard, the particular design of the three-dimensional imaging probe employed in the current study presents a reasonable compromise in terms of tomographic imaging geometry as it only provides 90^o^ of solid angular coverage around the illuminated side of the heart. Thus, indirect contributions from ultrasound waves affected by the lungs are largely avoided in the image reconstruction procedure.

In conclusion, we have shown the capabilities of volumetric optoacoustic cardiac imaging at an unprecedented time resolution. In combination with its unique advantages of high spatial resolution and functional and molecular optical contrast, the presented cardiac imaging approach anticipates a broad range of applications that require fast dynamic visualization of entire volumes currently unfeasible with other imaging modalities. Future experiments are tailored to provide functional and molecular assessments of healthy and diseased conditions of the mouse heart induced by transgenic or surgical means.

## Methods

### Experimental setup

The lay-out of the experimental setup is depicted in Fig. 1a. Basically, the setup is based on a custom-made spherical array of 256 piezoelectric elements (Imasonic, SaS, Voray, France) that efficiently collect the optoacoustic signals generated in a region close to the center of the sphere^35^,^36^. Optical illumination is provided by means of a fiber bundle inserted in a central cavity of the array. A short-pulsed wavelength-tunable laser with a pulse repetition rate of 50 Hz was used as light source (Innolas Laser GmbH, Krailling, Germany). The laser generated <10 ns duration pulses with per-pulse energy of approximately 20 mJ. The 256 optoacoustic signals were simultaneously acquired with a custom-made data acquisition system allowing operating at this pulse repetition rate (Falkenstein Mikrosysteme GmbH, Taufkirchen, Germany). The size and orientation of the transducer elements are essential to provide an acceptable signal to noise ratio (SNR) at this pulse repetition rate, especially if the energy per pulse must be kept below laser safety standards^37^. An agar block coupled with ultrasonic gel to the mouse skin and to the transducer elements is used in order to guarantee efficient ultrasound transmission. As both agar and the ultrasonic gel are transparent in the visible and near infrared, transmission of light is also ensured.

### *In vivo* mouse handling

An adult wild-type male CD1 mouse was used in the experiments. The hair in the chest area was removed with a shaving lotion (Fig. 1b). The mouse was anesthetized during the experiments with an isoflurane-medical air mixture (2% isoflurane, 0.8 L per min flow). Two experiments were conducted during anesthesia: one multispectral experiment, and another indocyanine-green injection experiment. In the first multispectral experiment, the mouse was imaged at four different wavelengths (730, 760, 800, and 850 nm), corresponding to different absorption values of oxygenated and deoxygenated hemoglobin. These wavelengths were chosen to elicit differences in the spectral absorption seen in the absorbance spectrum of oxygenated and deoxygenated hemoglobin, as shown in Fig. 3b. In a second experiment, cardiac imaging was performed during an intravenous tail-vein injection of 100 nmol indocyanine green (ICG). Several seconds of data were acquired at a single wavelength (800 nm) corresponding to the peak absorption of the probe. Animal experiments were conducted in full compliance with the institutional guidelines of the Institute for Biological and Medical Imaging and with approval from the Government District of Upper Bavaria.

### Data processing

The 256 optoacoustic signals corresponding to each frame consist of 2030 time samples acquired at a rate of 40 megasamples per second. The signals are first deconvolved with the impulse response of the individual piezoelectric elements (with central frequency 4 MHz and -6 dB bandwidth of 100%) and subsequently band-pass filtered with cut-off frequencies between 0.1 and 7.5 MHz. Three-dimensional images are reconstructed for a volume of interest of 14 × 14 × 12 mm^3^ with a parallel implementation of a back-projection formula, as described elsewhere^38^. An example of a three-dimensional view of a reconstructed image of the heart is displayed in Fig. 1c.

## Additional Information

**How to cite this article**: Deán-Ben, X. L. *et al.* High-frame rate four dimensional optoacoustic tomography enables visualization of cardiovascular dynamics and mouse heart perfusion. *Sci. Rep.* **5**, 10133; doi: 10.1038/srep10133 (2015).
