# Erratum: High-frame rate four dimensional optoacoustic tomography enables visualization of cardiovascular dynamics and mouse heart perfusion

**DOI:** 10.1038/srep13240

**Published:** 2015-08-21

**Authors:** Xosé Luís Deán-Ben, Steven James Ford, Daniel Razansky

Scientific Reports 5: Article number: 10133; 10.1038/srep10133published online: 07012015; updated: 08212015.

The original version of this Article contained a typographical error in the received date ‘11 November 2014’ which was incorrectly given as ‘11 November 2015.’ This error has now been corrected in the PDF and HTML versions of the Article.

In addition, the Supplementary Movies that accompany this study were omitted.

